# No evidence for an S cone contribution to acute neuroendocrine and alerting responses to light

**DOI:** 10.1016/j.cub.2019.11.031

**Published:** 2019-12-16

**Authors:** Manuel Spitschan, Rafael Lazar, Ebru Yetik, Christian Cajochen

**Affiliations:** 1 Department of Experimental Psychology, University of Oxford, Oxford OX2 6GG, UK; 2 Centre for Chronobiology, Psychiatric Hospital of the University of Basel (UPK), 4002 Basel, Switzerland; 3 Transfaculty Research Platform Molecular and Cognitive Neurosciences, University of Basel, 4002 Basel, Switzerland

## Abstract

Exposure to even moderately bright short-wavelength light in the evening can strongly suppress the production of melatonin and delay our circadian rhythm. These effects are mediated by the retinohypothalamic pathway, connecting a subset of retinal ganglion cells to the circadian pacemaker in the suprachiasmatic nucleus (SCN) in the brain. These retinal ganglion cells express the photosensitive protein melanopsin, rendering them intrinsically photosensitive (ipRGCs). But ipRGCs also receive input from the classical photoreceptors — the cones and rods. Here, in human participants, we examined whether the short-wavelength-sensitive (S) cones contribute to the neuroendocrine response to light by using stimuli which differed exclusively in the amount of S cone excitation by almost two orders of magnitude (ratio 1:83), but not in the excitation of long-wavelength-sensitive (L) and medium-wavelength-sensitive (M) cones, rods, and melanopsin. We specifically examined the S cones since the previously published action spectra for melatonin suppression [1,2] pointed to a possible role of S cones in addition to melanopsin. We find no evidence for a role of S cones in the acute alerting and melatonin-supressing response to evening light exposure.

## Teaser

Spitschan *et al.* examine the role of the S cones in the neuroendocrine response (melatonin suppression) to light in the evening using spectrally tailored lights with minimal impact on the other photoreceptors in the human retina.

## Main Text

**Figure 1:**
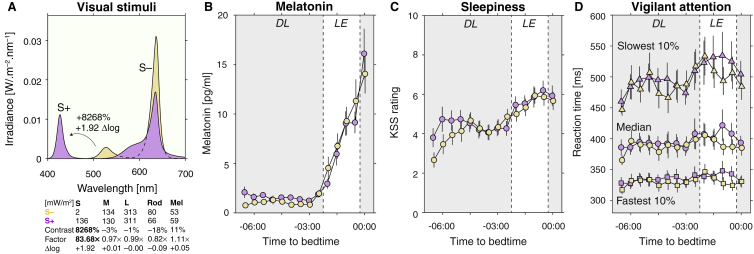
Experimental stimuli and their effects on salivary melatonin concentrations, subjective sleepiness and vigilant attention. (A) Spectral power distribution for the S-cone-isolating stimuli in peripheral presentation (annulus, inner ø =11°, outer ø = 58°), with minimal stimulation of L and M cones, rods, and melanopsin (S- = minimum S cone stimulation; S+ = maximum S cone stimulation, while retaining L and M cone, rod and melanopsin excitation). The difference in S cone stimulation can be specified as contrast (8268%), factor (83×), or log unit difference (+1.92 δlog). Numbers are calculated from the actual spectrum measured from the observer’s point of view. (B) Melatonin concentrations (mean ± 1 SEM), with characteristic increase in melatonin in the evening. No differential effect is observed in the S- and S+ conditions (Bayes factor [BF]: 0.71 ± 0.019). (C) Subjective sleepiness as measured using the Karolinska Sleepiness Scale (KSS), with characteristic increase in sleepiness in the evening (mean ± 1 SEM). No differential effect is observed in the S- and S+ conditions (BF: 0.43 ± 0.068). (D) Vigilant attention, as measured using simple reaction time (RT) to an auditory beep, showing median, fastest 10% and slowest 10% of RTs (mean ± 1SEM). No evidence is observed for an S cone influence on the slowest and fastest 10% RT, and only low anecdotal evidence in median RT (median RT BF: 1.4 ± 0.029; fastest 10% RT BF: 0.32 ± 0.031; slowest 10% RT BF: 0.44 ± 0.053). DL = dim light; LE = light exposure.

To probe the role of S cones in circadian responses to light, we generated a pair of stimuli providing either minimal S cone stimulation, S-, or maximal S cone stimulation, S+ (Figure 1A). The stimuli were designed to produce no differential stimulation of the L and M cones, the rods, and melanopsin. We employed a spectrally tuneable light source consisting of ten different LED lights, which were individually adjustable in intensity, thereby producing complex mixtures of light which differed in the amount of S cone stimulation by a factor of ∼83, or equivalently, ∼1.92 units at moderate photopic light levels (168–173 lux; Figure 1A). The S cones play an important role in colour vision, encoding the blue–yellow dimension of colour vision. As a consequence, our S-cone isolating stimuli appeared different in colour (but not luminance, or ‘brightness’), with S- corresponding to an orangish, and S+ corresponding to a pinkish colour (Figure S1E, published in Supplemental Information with this article online).

With these stimuli, we probed the human circadian timing system using acute melatonin suppression. Melatonin, which rises in concentration approximately two hours prior to habitual bedtime, can be strongly suppressed by short-wavelength light [1,2]. In an in-laboratory within-subject protocol under controlled lighting conditions, we found no difference in salivary melatonin production when participants (n = 15) were exposed to our two stimuli differing in S cone activation (Figure 1A) from 150 to 30 minutes prior to their habitual bedtime. While a change in light stimulus by almost two orders of magnitude (1:100) is known to move the neuroendocrine response to light from no response to saturation, a change of size in only the S cones produced no difference in the production of evening melatonin (Figure 1B; Bayes factor (BF) comparing full model with lighting condition as factor versus model without lighting condition: 0.71 ± 0.019). We also examined if our stimuli affected subjective sleepiness (measured using the Karolinska Sleepiness Scale; Figure 1C) and vigilant attention (measured using reaction time to beeps, averaged over ∼50 trials; Figure 1D). Neither sleepiness (BF: 0.43 ± 0.068) nor vigilant attention (median reaction time BF: 1.4 ± 0.029; fastest 10% reaction time BF: 0.32 ± 0.031; slowest 10% reaction time BF: 0.44 ± 0.053) were modulated by S cones alone.

Earlier studies located the peak spectral sensitivity for melatonin suppression near 460 nm [1,2], i.e. between the S cones and melanopsin. This spectral sensitivity cannot be described by a combination of L and M cones (‘luminance’) and a recent reanalysis of the data in [1] found they are best accounted for by melanopsin [3]. There is evidence for a possible minor contribution from the L and M cones decaying over time [4], but L and M cones are not necessary for melatonin suppression in people with red–green colour vision deficiencies affecting the L or M cones [5]. Finally, melatonin is suppressed by light in blind people with no cone/rod function at all [6]. Our finding that modulation of S cones alone cannot lead to appreciable neuroendocrine effects is consistent with this emerging picture.

In the primate retina, some ipRGCs receive positive, excitatory input from the L and M cones and negative, inhibitory synaptic input from the S cones [7]. Previous research also exploiting the method of silent substitution found paradoxical responses of the pupil to flickering S-cone-isolating stimuli [8]. Our findings suggest that the circuit responsible for pupil control may recruit different ipRGCs than those involved in circadian photoreception [9], adapts to differences in cone input, or may have different temporal integration properties downstream. In this issue, Mouland *et al.* [10] report circadian photoentrainment to light stimuli defined by S cone-opponent stimulation in mice, raising the interesting possibility that circadian phase shifts could be elicited by S cone-only stimulation.

Recent developments of lighting engineering and design have enabled the control of spectrum and intensity in the built environment. The lack of an S-cone mediated contribution to human neuroendocrine responses to light is a key piece in the puzzle to optimising lighting for human health and well-being.

## Supplemental Information

Document S1 Experimental procedures and one figure.

Table S1 Irradiance spectra.
