# Supplementary material for: No evidence for an S cone contribution to acute neuroendocrine and alerting responses to light
Source: Curr Biol. 2019 Dec 16;29(24):R1297–8. doi: 10.1016/j.cub.2019.11.031 (PMC6926470; doi:10.1016/j.cub.2019.11.031)
Supplement: Document S1 — Experimental procedures and one figure. [file mmc1.pdf]

Supplemental Information: No evidence for an S cone contribution to acute neuroendocrine and alerting responses to light

Manuel Spitschan[1, 2, 3 ¶, [0000-0002-8572-9268]], Rafael Lazar[2, 3, [0000-0001-7972-5634]], Ebru Yetik[2, 3, [0000-0002-4365-7235]] & Christian Cajochen[2, 3, [0000-0003-2699-7171]]

[1] *Department of Experimental Psychology, University of Oxford*, United Kingdom

[2] *Centre for Chronobiology, Psychiatric Hospital of the University of Basel (UPK)*, Switzerland

[3] *Transfaculty Research Platform Molecular and Cognitive Neurosciences, University of Basel*, Switzerland

[¶] Corresponding author: Dr Manuel Spitschan, manuel.spitschan@psy.ox.ac.uk

Acknowledgements

M.S. is supported by a Sir Henry Wellcome Fellowship (Wellcome Trust 204686/Z/16/Z) and a Junior Research Fellowship from Linacre College, University of Oxford. M.S. received equipment support from the John Fell Fund, University of Oxford.

Author contributions

Conceptualisation: M.S.; Methodology: M.S. and C.C.; Software: M.S.; Verification: M.S., R.L. and E.Y.; Investigation: M.S., R.L. and E.Y.; Resources: M.S. and C.C.; Writing – Original Draft: M.S.; Writing – Review & Editing: M.S., R.L. and C.C.

Declaration of Interests

M.S. is listed as an inventor on a US patent application (US Patent Application No. 14/852,001, “Robust Targeting of Photosensitive Molecules”) filed by the University of Pennsylvania (15 September 2015). M.S. is listed as an inventor on a UK priority patent application (GB1901723.5, "Determining Metameric Settings for a Non-linear Light Source”) filed by Oxford University Innovation Limited (7 February 2019).

Supplemental Experimental Procedures

Participant characteristics. Seventeen male participants (n=17) aged 18-35 were recruited to participate in the study (mean age±1SD: 24.1±2.72 years). Our participants were screened for sleep disruption (>5 on Pittsburgh Sleep Quality Index, PSQI, [S1]), extreme morningness or eveningness (>27 or <11 on modified Horne & Östberg questionnaire, [S2]), depressive symptoms (>27 on Center for Epidemiologic Studies Depression scale, CES-D, [S3]), alcohol use disorder (>19 on Alcohol Use Disorders Identification Test, AUDIT, [S4]), abnormal colour vision (assessed with Hardy-Rand-Rittler [HRR] plates), and visual acuity (at least 20/40 assessed using Snellen chart).

Data exclusion and missing data. We had to exclude two of the 17 participants in our analysis. Our analyses of the melatonin, sleepiness (KSS), and vigilant attention (auditory RT) data do not include these two participants. One participant was excluded due to stimulus mistiming (melatonin concentrations never exceeded 5 pg/mL). The other one was excluded due to implausible high melatonin levels (>300 pg/mL) in one session, possibly pointing to contamination of the samples.

In some samples of the remaining participants, the assays returned implausibly high melatonin samples (e.g. >30 pg/mL six hours before habitual bedtime), in which case we detected and removed outliers across participants but within-condition using the iterative generalized extreme Studentized deviate test for outliers (implemented in MATLAB's `isoutlier` function). This affected 20 samples of the total 420 samples (15 participants × 2 sessions × 14 samples per sessions), leading to an exclusion rate of <5% of samples.

Stimulus design and delivery. Visual stimuli were generated using a 10-primary LED-based light source (SpectraTune LAB, Ledmotive Technologies S.L, Barcelona, Spain) imaged onto a diffusing surface with independent 12-bit (4096 levels, including off) software control over the spectral emittance over each primary. Eight of the 10 primaries were relatively narrowband (427±16 nm [peak wavelength±FWHM at 100% intensity], CIE 1931 xy chromaticity: (0.17, 0.02); 445±20 nm, (0.17, 0.03); 465±24 nm (0.14, 0.07), 474±30 nm (0.13, 0.12), 504±31 nm (0.11, 0.58), 522±34 nm (0.19, 0.71), 636±19 nm (0.70, 0.30), 659±19 nm (0.72, 0.28). Two additional primaries were broadband LEDs: lime (558±120 nm, (0.43, 0.54)) and orange (596±83 nm, (0.57, 0.52)). A mask was placed in front of the diffusing surface, so as to provide an annular region with an outer diameter of 20 cm and an inner diameter of 3.5 cm, viewed at a viewing distance of 18 cm from a chin rest (annulus inner diameter: ~11°, outer diameter ~58°), thereby providing peripheral stimulation appropriate for circadian responses to light [S5].

We generated our S-cone-selective stimuli using the method of silent substitution [S6,S7]. In the method of silent substitution, pairs of spectra are generated as mixtures of the ten primaries lights which produce a difference in only one photoreceptor class (in this case, the *stimulated* S cones), while there is no difference in the other photoreceptors (in this case, the *silenced* L and M cones, rods, and melanopsin). This method has previously been used to examine the effect of melanopsin-only differences in lighting on melatonin suppression [S8, S9] (but has a long history in vision science, see [S7]).

To produce calibrated stimuli, we first measured the spectral radiance of each LED independently at 19 intensity levels (spaced at 5% increments from 5% to 100%, where 100% is maximum intensity) using a spectroradiometer (spectroval 1511, JETI Instruments GmbH, Jena, Germany). We addressed the typical changes in spectrum with increasing intensity by relying on an interpolation-based forward model of our primaries (interpolating at unmeasured primary settings). Using this model, we generated two sets of settings for our primaries which would have the feature that they yielded maximum differential stimulation on the S cones, with minimal change in L and M cone, rod and melanopsin stimulation. These settings were simultaneously found using constrained minimisation routines implemented in MATLAB (`fmincon` SQP solver with global optimisation; 1000 trial points). In this procedure, we used the cone, rod and melanopsin spectral sensitivities [S10] comprising the 10° Stockman-Sharpe cone fundamentals [S11], the CIE V'(λ) function for the rods, and the standard curve for melanopsin [S12]. Irradiance spectra measured in the corneal plane from the observer's point of view are given in Table S1.

We quantified the difference in S cone excitation by calculating the Weber contrast (percentage change in S cones from S– to S+) and as a factor (ratio of S cone excitation between S+ and S–). Both numbers are equivalent representations of the stimulus change. We report both numbers for completeness. For clarity, in the main text, we round the factor down to the nearest integer so as not to overstate the stimulus change.

We achieved a stimulus with a difference of 8268% (factor 83.68×), or equivalently almost two log units (~1.92 log difference), in S cone stimulation, with minimal stimulation of L and M cones, rods and melanopsin. The photopic illuminances were 168 lux for the S– condition (0.48, 0.26; 'orange' appearance) and 173 lux for the S+ condition (0.61, 0.37; 'pink' appearance). The melanopic irradiance was 59 mW/m^2 for the S+ condition and 53 mW/m^2 for the S– condition. These background radiances correspond to moderate photopic light levels.

Validating the spectra from this optimisation procedure, our stimuli demonstrated excellent silencing for the L and M cones (Fig. 1; –3% L cone contrast, –1% M cone contrast), and very good silencing for rods (–18%) and melanopsin (+11%), while providing an almost two log unit difference S cone stimulation. It is unlikely that these small nominal differences produce a meaningful physiological difference, given the very large and to our knowledge unparalleled difference in S cone stimulation.

Protocol. The study took place in a dedicated light-, temperature- and humidity-controlled apartment comprising a double-room as well as a dedicated bathroom (see Appendix A in [S13] for photograph). Upon arrival (30 minutes prior to protocol start), participants gave a urine sample for drug test (multi-drug panel test for AMP, BZD, COC, MOR/OPI, MTD and THC; exclusion if positive; nal von minden, Den Haag, Netherlands) and accommodated to the laboratory. Then, the protocol began, lasting from 6.5 hours before habitual bedtime to habitual bedtime. Every 30 minutes, participants completed an alertness assessment using a simple auditory reaction time task, the Karolinska Sleepiness Scale (KSS), and gave a saliva sample using Salivettes in dim light provided by room illumination (photopic illuminance in the corneal plane <8 lux). From 2.5 hours to 0.5 hours prior to the habitual bedtime,

participants were either exposed to the S– or S+ stimuli in 20-minute sections under steady fixation, yielding a total of 80 minutes of light exposure to the experimental stimuli. Between the 20-minute sections, participants completed the questionnaire, performed the PVT and gave the saliva sample under the dim <8 lux lighting. This protocol balanced feasibility of light exposure with the possibility that cone responses might adapt during long-term light exposure.

Fixation and eye opening were verified using a video-based head-mounted eye tracker (Pupil Labs GmbH, Berlin, Germany). Participants had access to water throughout the experiment but no food or other drinks. Participants were allowed to spend their time reading, studying, playing Nintendo GameBoy (illuminance at cornea <8 lux), or other activities not involving additional light exposure. Smartphones and other electronic devices were removed from the experiment suite. All experiments took place between November 2018 and June 2019. All sessions took place one week from another and condition order was randomised between participants. From one week prior to the experiment to the second session, participants were instructed to adhere to regular bedtimes (±30 minutes) and wore actigraphy devices (Condor Instruments, São Paolo, Brasil). On the day of the experiment, participants were asked to refrain from caffeine consumption after noon.

Salivary melatonin. Saliva samples (at least 1 mL) were collected at 30-minute intervals using Salivettes (Sarstedt AG, Sevelen, Switzerland), which were immediately centrifuged and frozen at -20° for later assay. Melatonin was measured using a direct double-antibody radioimmunoassay previously validated against serum levels (minimum detectable dose 0.2 pg/mL; Bühlmann Laboratories AG, Allschwil, Switzerland) [S14].

Vigilant Attention. Vigilant Attention was measured using a custom-made simple auditory reaction time task programmed in Psychtoolbox and MATLAB (The Mathworks, Natick, MA). Participants were presented with a tone emitted from a loudspeaker and were instructed to press as quickly as possible to the tone using a PlayStation-like gamepad. ISI was randomly set to 5-8 seconds. Median reaction times were calculated from 50 trials.

In-laboratory light questionnaire. Participants were asked to rate or respond to various aspects of the light exposure using a 6-question, 7-item Likert scale questionnaire. This questionnaire was administered in German. The questions were about the *comfort of light* (“Allgemein ist das Licht angenehm”; überhaupt nicht [S1] – sehr stark [S7]), the *perceived brightness* (“Wie empfinden Sie die Helligkeit des Lichtes?”; sehr dunkel [S1] – sehr hell [S7]), *light level preference* (“Ich hätte es lieber …”; deutlich dunkler [S1] – deutlich heller [7]), *glare* (“Dieses Licht blendet mich”; überhaupt nicht [S1] – sehr stark [S7]), *the perceived colour temperature* (“Wie empfinden Sie die Lichtfarbe?”; sehr kalt [S1] – sehr warm [7]) and *general well-being* (“Wie fühlen Sie sich im Moment?”; unwohl [S1] – wohl [S7]).

Karolinska Sleepiness Scale (KSS). We used the *German* version of the Karolinska Sleepiness Scale (“Bitte bewerten Sie Ihre Müdigkeit” (“sehr wach” [S1], “wach” [S3], “weder wach noch müde” [S5], “müde, aber keine Probleme, wach zu bleiben” [S7], “sehr müde, große Probleme, wach zu bleiben, mit dem Schlaf kämpfend” [S9]).

Statistical analysis. We modelled our data using a linear mixed-effects model, modelling subjects as a random-effects, and condition (S+ or S–) and sample number (with sample #14 corresponding to habitual bedtime) as fixed effects, along with the interaction between condition and sample. In Wilkinson-Rogers notation, the full model (M_1) is specified as

```
outcome ~ Sample + Condition + (1|participant).
```

The null model (M_0, no effect of S cone manipulation) is specified as

```
outcome ~ Sample + (1|participant).
```

To estimate the evidential strength for an S cone manipulation, we calculated Bayes factors (BF) using the R package 'BayesFactor' (version 0.9.12-4.2) [S15-S17]. Compared to traditional null hypothesis significance testing, this approach allows for assessing the evidential strength of competing models. Bayes factors specify the ratio of the marginal likelihood of two competing models. We used standard scales for interpreting the Bayes factor [S18] and considered both the full data (all data points) and the data points only during the light exposure. The resulting Bayes factors are given below.

**Variable**	**BF (all data points)**	**Evidential strength [18]**	**BF (light exposure)**	**Evidential strength [18]**
Salivary melatonin concentration	0.22±0.0134	Moderate evidence (M_0)	0.71±0.019	Anecdotal evidence (M_0)
Sleepiness	1.01+0.0143	Anecdotal evidence (M_1)	0.43±0.068	Anecdotal evidence (M_0)
RT (Median)	1.59±0.0339	Anecdotal evidence (M_1)	1.4±0.029	Anecdotal evidence (M_1)
RT (Fastest 10%)	0.31±0.0214	Anecdotal evidence (M_0)	0.32±0.031	Anecdotal evidence (M_0)
RT (Slowest 10%)	0.34±0.0172	Anecdotal evidence (M_0)	0.44±0.053	Anecdotal evidence (M_0)
Visual comfort	0.12±0.0194	Moderate evidence (M_0)	0.59±0.019	Anecdotal evidence (M_0)
Brightness	0.2±0.0093	Moderate evidence (M_0)	0.23±0.042	Moderate evidence (M_0)
Preference	0.27±0.02	Moderate evidence (M_0)	0.62±0.018	Anecdotal evidence (M_0)
Glare	0.12±0.0464	Moderate evidence (M_0)	0.5±0.02	Anecdotal evidence (M_0)
Colour temperature	17.73±0.02	Strong evidence (M_1)	452035.91±0.023	Extreme evidence (M_1)
General well-being	0.12±0.0792	Moderate evidence (M_0)	0.23±0.043	Moderate evidence (M_0)

Ethical approval. This study was approved by the cantonal ethics commission (Ethikkommission Nordwest- und Zentralschweiz, PB_2018-00164 – 280/90) and was conducted in accordance with the Swiss law and according to the Declaration of Helsinki.

Supplemental Figures

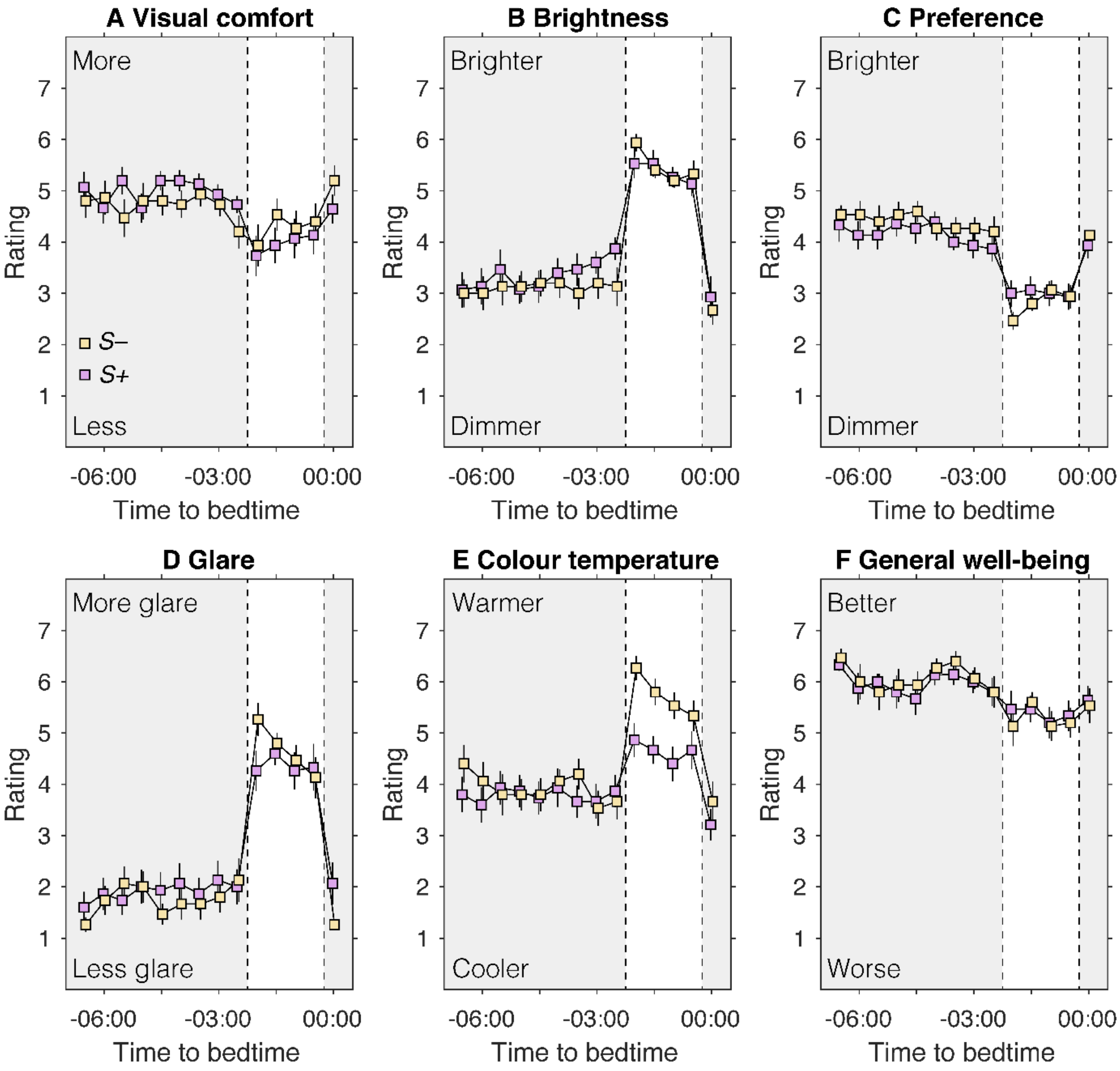

Figure **S1**. Rating results for visual comfort (***A***), perceived brightness (**B**), light level preference (***C***), glare (***D***), perceived colour temperature (***E***), and general well-being (***F***). Details about questions are given in the text. During light exposure, lighting condition (S+ or S–) did not strongly affect visual comfort (BF: 0.59±0.019 for full vs. null model), perceived brightness (BF: 0.23±0.042 for full vs. null model), light level preference (BF: 0.62±0.018 for full vs. null model), glare (BF: 0.5±0.02 for full vs. null model), or general well-being (BF: 0.23±0.043 for full vs. null model), but strongly affected perceived colour temperature (BF: 452035.91±0.023 for full vs. null model).

Supplemental References

S1. Buysse, D.J., Reynolds, C.F., 3rd, Monk, T.H., Berman, S.R., and Kupfer, D.J. (1989). The Pittsburgh Sleep Quality Index: a new instrument for psychiatric practice and research. Psychiatry Res *28*, 193-213.

S2. Adan, A., and Almirall, H. (1991). Horne & Östberg morningness-eveningness questionnaire: A reduced scale. Pers Individ Dif *12*, 241-253.

S3. Radloff, L.S. (1977). The CES-D Scale. Applied Psychological Measurement *1*, 385-401.

S4. Saunders, J.B., Aasland, O.G., Babor, T.F., de la Fuente, J.R., and Grant, M. (1993). Development of the Alcohol Use Disorders Identification Test (AUDIT): WHO Collaborative Project on Early Detection of Persons with Harmful Alcohol Consumption--II. Addiction *88*, 791-804.

S5. Adler, J.S., Kripke, D.F., Loving, R.T., and Berga, S.L. (1992). Peripheral vision suppression of melatonin. J Pineal Res *12*, 49-52.

S6. Spitschan, M., and Woelders, T. (2018). The method of silent substitution for examining melanopsin contributions to pupil control. Front Neurol *9*, 941.

S7. Estevez, O., and Spekreijse, H. (1982). The "silent substitution" method in visual research. Vision Res *22*, 681-691.

S8. Allen, A.E., Hazelhoff, E.M., Martial, F.P., Cajochen, C., and Lucas, R.J. (2018). Exploiting metamerism to regulate the impact of a visual display on alertness and melatonin suppression independent of visual appearance. Sleep *41*.

S9. Souman, J.L., Borra, T., de Goijer, I., Schlangen, L.J.M., Vlaskamp, B.N.S., and Lucassen, M.P. (2018). Spectral Tuning of White Light Allows for Strong Reduction in Melatonin Suppression without Changing Illumination Level or Color Temperature. J Biol Rhythms *33*, 420-431.

S10. CIE (2018). CIE S 026/E:2018: CIE System for Metrology of Optical Radiation for ipRGC-influenced Responses To Light, (Vienna, Austria: CIE Central Bureau).

S11. CIE (2006). CIE 170-1: Fundamental Chromaticity Diagram with Physiological Axes – Part 1: Definition of CIE 2006 Cone Fundamentals, (Vienna, Austria: CIE Central Bureau).

S12. Lucas, R.J., Peirson, S.N., Berson, D.M., Brown, T.M., Cooper, H.M., Czeisler, C.A., Figueiro, M.G., Gamlin, P.D., Lockley, S.W., O'Hagan, J.B., et al. (2014). Measuring and using light in the melanopsin age. Trends Neurosci *37*, 1-9.

S13. Spitschan, M., Stefani, O., Blattner, P., Gronfier, C., Lockley, S.W., and Lucas, R.J. (2019). How to report light exposure in human chronobiology and sleep research experiments. Clocks Sleep *1*, 280-289.

S14. Weber, J.M., Schwander, J.C., Unger, I., and Meider, D. (1997). A direct ultrasensitive RIA for the determination of melatonin in human saliva: comparison with serum levels [Abstract]. Sleep Res *26*, 757.

S15. Morey, R.D., and Rouder, J.N. (2011). Bayes factor approaches for testing interval null hypotheses. Psychol Methods *16*, 406-419.

S16. Morey, R.D., Rouder, J.N., Pratte, M.S., and Speckman, P.L. (2011). Using MCMC chain outputs to efficiently estimate Bayes factors. J Math Psychol *55*, 368-378.

S17. Rouder, J.N., Speckman, P.L., Sun, D., Morey, R.D., and Iverson, G. (2009). Bayesian t tests for accepting and rejecting the null hypothesis. Psychon Bull Rev *16*, 225-237.

S18. Jeffreys, H. (1961). Theory of probability, (Oxford, England: Oxford University Press).
